# An authenticity survey of herbal medicines from markets in China using DNA barcoding

**DOI:** 10.1038/srep18723

**Published:** 2016-01-07

**Authors:** Jianping Han, Xiaohui Pang, Baosheng Liao, Hui Yao, Jingyuan Song, Shilin Chen

**Affiliations:** 1 Institute of Medicinal Plant Development, Chinese Academy of Medicinal Science &Peking Union Medicinal College, Beijing 100193, P.R. China; 2 Institute of Chinese Materia Medica, China Academy of Chinese Medical Sciences, Beijing 100700, P.R. China

## Abstract

Adulterant herbal materials are a threat to consumer safety. In this study, we used DNA barcoding to investigate the proportions and varieties of adulterant species in traditional Chinese medicine (TCM) markets. We used a DNA barcode database of TCM (TCMD) that was established by our group to investigate 1436 samples representing 295 medicinal species from 7 primary TCM markets in China. The results indicate that ITS2 barcodes could be generated for most of the samples (87.7%) using a standard protocol. Of the 1260 samples, approximately 4.2% were identified as adulterants. The adulterant focused on medicinal species such as Ginseng Radix et Rhizoma (Renshen), Radix Rubi Parvifolii (Maomeigen), Dalbergiae odoriferae Lignum (Jiangxiang), Acori Tatarinowii Rhizoma (Shichangpu), Inulae Flos (Xuanfuhua), Lonicerae Japonicae Flos (Jinyinhua), Acanthopanacis Cortex (Wujiapi) and Bupleuri Radix (Chaihu). The survey revealed that adulterant species are present in the Chinese market, and these adulterants pose a risk to consumer health. Thus, regulatory measures should be adopted immediately. We suggest that a traceable platform based on DNA barcode sequences be established for TCM market supervision.

Counterfeit drugs are frequently sold on the market, resulting in adverse effects, drug resistance, and death^1^,^2^,^3^. The proportion of counterfeit drug sales in developing countries is approximately 10%, and the proportion may be greater than 50% when considering medicines purchased online^4^,^5^,^6^. Drug counterfeiting causes health problems, particularly in developing countries where drug regulatory systems are weak or ineffective^7^. Although organizations are working to develop methods to protect the drug supply chain, counterfeit drug use has dramatically increased in recent years. Each week, new cases of counterfeit medicines are reported around the world^8^. According to numerous official sources, the proportion of counterfeit medicines in African countries has reached 80%^9^. Because of a lack of suitable identification methods, the number of reported cases of counterfeit medicine seems to be rising^9^.

Traditional Chinese medicine (TCM) plays an important role in disease prevention and treatment, and research has demonstrated the clinical efficacy of TCM against certain diseases for which conventional therapy is ineffective or has associated side effects^10^. In recent years, there has been a huge increase in the use of herbal products; however, there are also numerous reports of adulterant herbal medicine use in many developing countries, which poses a major public health risk. The World Health Organization (WHO) defines a counterfeit product as one that is mislabelled deliberately and fraudulently with respect to its identity or source. Crude materials provide the basis for genuine drug production, but recently, there have been many alarming reports of counterfeit or adulterant drugs that caused life-threatening poisonings. For example, a type of tea adulterant containing *Adenostyles alliaria*e caused serious liver disease after long-term use^11^,^12^. Adulterant tea mixed with *Illicium anisatum* (which contains neurotoxic substances)^13^ and cases of toxicity caused by *Aconitum*^14^ and *Datura metel*^15^ have also been reported. Moreover, approximately 50% of artesunate (extracted from *Artemisia annua* L.) tablets sampled in Southeast Asia were reported to be counterfeit^16^; Severe kidney damage caused by adulteration with *Aristolochia* species is frequently reported^17^,^18^,^19^, as a result of aristolochic acid toxicity. Song showed that 60% of commercial *Rhodiola* products are adulterants, which indicates a potential safety issue^20^. All of these life- threatening poisoning cases threaten the safe use of TCM. As a result, the detection of adulterant drugs is becoming a growing challenge^8^.

Traditional identification methods recognize materials by their morphological characteristics, and these methods primarily depend on human expertise. However, in some cases, it is extremely difficult for taxonomists to definitively identify plant genera, such as *Crataegus* and *Salix.* Chemical analyses, such as high-performance liquid chromatographic- mass spectrometric (HPLC–MS)^21^, near-infrared spectroscopy (NIRS)^22^ and liquid chromatography-mass spectrometry (LC–MS assays)^23^, can be used to detect chemical compositions to identify adulterant products. However, none of these methods alone can definitively identify closely related species that share remarkably similar morphological characteristics and chemical profiles. These techniques produce only indirect evidence of fraud and cannot definitively determine the identity of the given species. Therefore, there is an urgent need for rapid and simple identification procedures for the rapid inspection of raw herbal materials.

DNA barcoding is a new molecular diagnostic technology that was first proposed by Canadian zoologist Paul Hebert in 2003, and it identifies species by using a recognized standard, short genomic sequence^24^. DNA barcoding provides consistent and reliable results regardless of the age, plant part, or environmental factors of the sample^25^. Researchers can evaluate species information accurately by analysing DNA sequences. Other investigators have suggested that a global DNA barcode revolution would become a “big science” research programme after the human genome project^26^, and Miller published “the Renaissance of DNA barcode and taxonomy” in PNAS^27^. This approach has been repeatedly reported in academic journals (e.g., Nature, Science) and in media outlets (e.g., National Geographic News, The New York Times) stating that DNA barcode technology has become a global innovation for academic research on biological taxonomy. Chen *et al.* have analysed more than 6600 plant samples belonging to 4800 species from 753 distinct genera by using the chloroplast regions *psbA-trnH, matK, rbcL, rpoC1, ycf5* and the nuclear loci ITS and ITS2. These investigators suggested that the internal transcribed spacer (ITS) fails to be amplified and sequenced in most samples and that ITS2 is the most suitable locus for DNA barcoding research, followed by *psbA-trnH* as a complementary region^28^. By using an ITS2 + *psbA*-*trnH* two-loci barcode combination, our group developed a TCM barcode platform, called the Traditional Chinese Medicine Database (TCMD)^29^, which contains 78,847 barcodes belonging to 23,262 medicinal species listed in the Chinese, European, Indian, Japanese, Korean and American Herbal Pharmacopoeias^30^,^31^,^32^,^33^,^34^,^35^. There are more than three samples per species in this database^29^. At present, the TCMD is the largest DNA barcode database of medicinal materials. The TCMD also contains the DNA barcoding standard operating procedure (SOP) and provides bioinformatics tools to assist in data analysis for researchers in the herbal identification industry. The TCMD can be accessed at http://www.tcmbarcode.cn/en/.

In this study, we investigated the proportions and varieties of adulterant medicine in herb markets with the aim of protecting consumers from health risks associated with herbal product substitution and contamination by using a standard DNA barcoding method. A total of 1436 raw herbal samples representing 295 medicinal species were collected from the 7 primary markets in China. The advantages and limitations of DNA barcoding for the authentication of complex TCM materials by using the TCMD database are also discussed. Additional details are described in subsequent sections.

## Results

### Efficiency of PCR amplification and sequencing

Of the 1436 samples, 176 (12.26%) could not be successfully amplified and sequenced, primarily cortex and fungal medicinal species. The failure rates of cortex and fungi medicinal species were approximately 21/93 (22.6%) and 5/23 (21.7%), respectively. The unamplified species were Magnoliae Officinalis Cortex (Houpo), Periplocae Cortex (Xiangjiapi), Phellodendri Chinensis Cortex (Huangbo), Fraxini Cortex (Qinpi) and Polyporus (Zhuling). In contrast, stem and folium medicinal species were easily amplified, with failure rates of approximately 3.1% and 5.1%, respectively. There was difficulty with the DNA extraction for 77 radix et rhizome species (15.0%), including Asteris Radix et Rhizoma (Ziwan) and Gastrodiae Rhizoma (Tianma); only 1/4 or 1/5 of the sequences were generated from these species. The amplification of the ITS2 sequences failed for approximately 41/451 (9.1%) of the fruit and seed samples, including 6/7 of the Chebulae Fructus (Hezi) (85.7%), 6/7 of the Aurantii Fructus Immaturus (Zhishi) (85.7%), and 3/5 (60.0%) of the Alpiniae oxyphyllae Fructus (Yizhi).

### Proportions and varieties of adulterant species revealed by the TCMD

BLAST1 was used to estimate the reliability of species identification by the TCMD^29^. We searched the 1260 ITS2 sequences generated in this study in the TCMD database. The ITS2 region results indicated that 4.2% of the sample names were not in accordance with the commercial name (Table 1).

**Figure 1:**
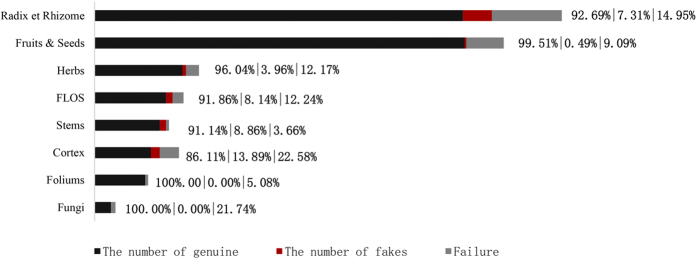
The adulterant rate from different samples of medicinal materials, including the radix et rhizome, fruits and seeds, herbs, flos, stems, cortex, foliums, and fungi.

No adulterants were found in the fungal and folium samples (Fig. 1). All of the 18 fungi and 56 folium samples were authenticated. Approximately 13.9% of the cortex samples were found to be adulterant, including the Albiziae Cortex (Hehuanpi), Pseudolaricis Cortex (Tujingpi) and Acanthopanacis Cortex (Wujiapi) from different markets. Of the 410 total sequences generated from the fruit and seed samples, only 2 were adulterants, including one sample of Sojae Semen Praeparatum (Dandouchi) and one sample of Alpiniae oxyphyllae Fructus (Yizhi). The adulterant rate and the failed amplification of flos samples were approximately 8.1% and 12.2%, respectively. Of the 438 total ITS2 sequences generated from the radix et rhizome samples, approximately 7.31% were adulterant.

**Figure 2:**
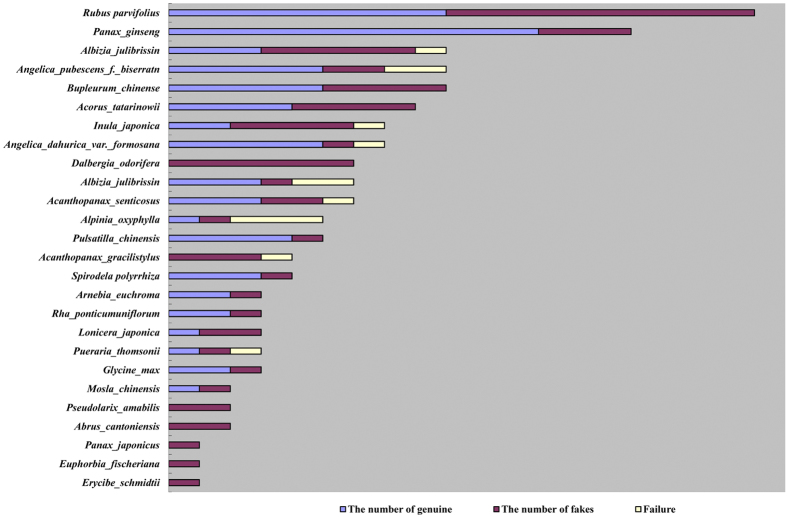
The adulterant rate observed for 26 medicinal plants.

Of the 295 medicinal species in this study, 198 could be amplified successfully and were validated, including species that are commonly used in TCM, such as Fritillariae cirrhosae Bulbus (Chuanbeimu), Rhei Radix et Rhizoma (Dahuang), Angelicae Sinensis Radix (Danggui), Codonopsis Radix (Dangshen), Saposhnikoviae Radix (Fangfeng), Glycyrrhizae Radix Rhizoma (Gancao), and Polygoni multiflorum Radix (Heshouwu), and the other 97 varieties exhibited failed amplification and adulterants to some extent. The adulterants included species such as Ginseng Radix et Rhizoma (Renshen), Radix Rubi Parvifolii (Maomeigen), Dalbergiae odoriferae Lignum (Jiangxiang), Acori Tatarinowii Rhizoma (Shichangpu), Inulae Flos (Xuanfuhua), Lonicerae Japonicae Flos (Jinyinhua), Acanthopanacis Cortex (Wujiapi) and Bupleuri Radix (Chaihu). The original species of Albiziae Cortex (Hehuanpi) was *Albizia julibrissin*, but five of the 9 Albiziae Cortex (Hehuanpi) samples were found to be derived from the Cortex of *Albizia kalkora* Prain (Shanhehuanpi) (Fig. 2), 3 of the 15 Ginseng Radix et Rhizoma (derived from *Panax ginseng*) samples were found to be Panacis quinquefolii Radix (derived from *Panax quinquefolius*), and 10 of the 19 Radix Rubi Parvifolii (Maomeigen) samples were Cirsii Japonici Heiba (Daji), Rosae Chinensis Flos (Yuejihua) or the root of *Rubus alceaefolius*. Two Lonicerae Japonicae Flos (derived from *Lonicera japonica*) samples were found to be Lonicerae Flos (derived from *Lonicera macranthoides)*. Of the 4 Acanthopanacis Cortex (Wujiapi) samples, 1 was an amplification failure, 2 were identified as Periplocae Cortex (Xiangjiapi) and one sample was derived from the Cortex of *Eleutherococcus giraldii* Harms (Hongmaowujiapi). All 6 of the Dalbergiae odoriferae Lignum (Jiangxiang) samples were adulterant, and they were identified as Sappan Lignum (derived from *Caesalpinia sappan*). In total, 53 samples were adulterants, and for 9 samples, the exact species could not be determined (Table 1).

### Survey of 7 herb markets

The 7 herb markets investigated in this study included Guangxi Yulin (GX), Hebei Anguo (AG), Henan Yuzhou (HN), Anhui Bozhou (BZ), Chongqing Cuqimeng (CQ), Guangdong Qingping (QP), and Sichuan Hehuachi (HUC). With the exception of HUC, different types of adulterant medicine were found at all of the herb markets, with percentages ranging from 3.7% in AG to 13.3% in QP. Of the 176 unamplified samples, CQ had the highest rate, at approximately 27.4%, and the rates for QP, HN and GX were approximately 18.9%, 16.8% and 13.5%, respectively. HUC had the lowest rate.

## Discussion

### Advantages and limitations of DNA barcoding in the quality analysis of crude TCM materials

DNA barcoding is a universal method that can be used to develop an international standard for product identification. At the Fourth International Barcode of Life Conference, a three-loci barcode (*matK* + *rbcL* + *psbA−trnH*) was suggested for plant identification. Chen *et al.* suggested ITS2 as a preferred barcode for medicinal plants^28^. Han *et al.* also showed that ITS2 is suitable for identifying medicinal samples^36^. In the present study, ITS2 was shown to be a very promising and effective tool for assessing adulterant in TCM markets. Of the 1260 ITS2 sequences generated, 4.2% were adulterants. Only 1 of the 7 herb markets provided authentic products with no adulterants.

The use of DNA barcoding to identify commercialized medicinal plants in southern Morocco suggests that a reference barcoding database should contain an adequate number of sequences from different locations^37^. Previous studies have defined the uncertainties of assigning unknown herbal products with incomplete reference barcode databases in GenBank and BOLD. One of the goals of the Herb-BOL (barcode of life) research programme was to build an herbal barcode library that covered all 1800 known medicinal species used in commercial products. Because of the importance of authenticating medicinal plant materials, it is vital to develop an exclusive, extensive herbal database^25^. The GenBank database (http://www.ncbi.nlm.nih.gov/genbank/) is possibly one of the largest sequence databases and is one of the most frequently used databases for species identification. An unknown DNA sequence can be rapidly compared to known species sequences with the BLAST program^38^. However, at present, many medicinal sample sequences are not adequately represented in GenBank, and in some cases investigators could only declare results at the genus level based on sequence similarity. The TCMD is a barcode database that is exclusively devoted to medicinal species, and it contains 23,262 medicinal and closely related species, including adulterants and substitutions. The TCMD covers almost all the medicinal materials listed in herbal pharmacopoeias from around the world, including China, Europe, India, Japan, Korea and the United States. Currently, the TCMD is the largest DNA barcode database of medicinal materials in the world^29^. Thus, the TCMD platform is the most suitable for the rapid screening of crude medicinal materials. The establishment of the TCMD has greatly improved the resources available for medicinal species identification.

Given that some medicinal samples are heavily processed and that some artificial adulterant samples do not contain DNA, DNA barcoding is not sufficient to confirm the identity of any given sample. In the current investigation, we found that at least 50% of the medicinal materials on the market have been fumigated with sulphur to extend the storage time and prevent insect infestation and mildew. In some cases, samples treated with sulphur, such as *Lycium barbarum* and *Dioscorea opposite,* appeared very clean and bright in colour and could be sold at a high price. This factor may also affect the amplification efficiency of the sample. In addition, many herbs contain secondary compounds such as polysaccharides, pigments and others. We washed the precipitants with wash buffer three times to remove sticky residues before extraction, but some of the residues could not be removed, which could also make it difficult to extract DNA from these samples. Approximately 12.26% of the samples evaluated in this study could not be successfully amplified and sequenced.

DNA barcoding is an efficient tool for the identification of herbs and for the determination of various adulterants. However, DNA barcoding does not currently yield information regarding the concentration of active ingredients. Thus, DNA barcoding cannot be used to determine whether medicinal samples meet pharmacopoeia standards. In other words, DNA barcoding can be used to establish herbal authenticity but cannot be used to evaluate herbal quality. This drawback indicates that a combination of DNA barcoding and chemical analysis is necessary for a comprehensive quality assessment of herbal samples. HPLC has been used for the differentiation of accessions collected from different geographic regions. DNA barcoding has been used for the differentiation of inter- and intraspecific variations and to detect adulterations. Attempts have also been made by the author to match the results of DNA barcoding to the chemical analysis techniques of Salvia L^39^.

### Building a traceable platform for traditional Chinese medicine using DNA barcoding

In many developing countries, the introduction of herbal medicine products into the marketplace is not adequately monitored. Genuine (Daodi) herbs are usually considered to be high-quality medicinal materials that are produced in the Daodi area. However, because many genuine medicinal plants are transported to other places, their characteristics will be changed. In TCM markets, many sellers advertise that their herbs come from the genuine area, but there are no methods to evaluate genuine characteristics. Furthermore, herbal medicine contamination is higher because of the lower stringency of the rules and regulations governing the quality of these herbs in different countries^40^,^41^. In the present study, a survey of TCM markets identified approximately 4.2% of the samples as adulterants. Such adulterant incidents will only increase if measures are not taken to prevent them. Thus, it is necessary to build a traceable platform to ensure the safe use of TCM.

**Figure 3:**
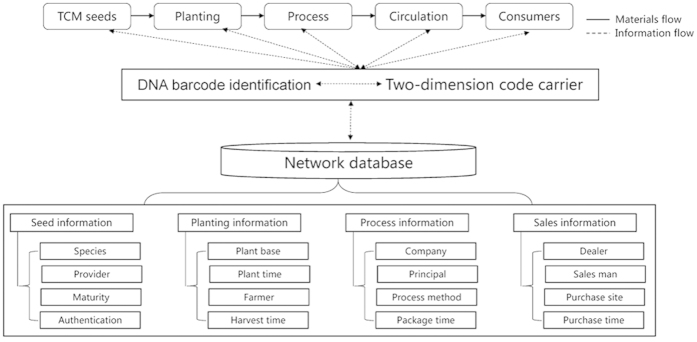
The framework for the traceability platform of traditional Chinese medicine based on DNA barcoding.

At present, DNA barcode technology is the best technology for providing traceability. Liu *et al.*^42^ successfully converted DNA barcoding sequences into two-dimensional barcodes (2D-barcodes). In addition, our research group has developed an automated process that converts DNA barcode sequences into 1D- and 2D-barcodes. Other information, including planting, processing and additional consumer information, can also be databased and converted into a 2D-barcode. Smartphones can be used as 2D-barcode readers so that consumers can conveniently scan samples to access information. This type of traceability system would not only help to manage TCM authentication but would also provide a valuable tool to improve TCM quality. Consumers could obtain all the information regarding a commercial TCM that was on the market, including planting, production, processing and circulation information, by scanning the 2D-barcode on the package. A workflow outlining such a system is shown in Fig. 3. In view of the above information, the establishment of a traceability system for TCM based on DNA barcode sequences is urgently needed.

### The future of DNA barcoding

Traditionally, commonly used identification methods require special skills acquired through extensive experience; thus, only experts can identify taxa accurately. The current study showed that ITS2 sequences could be used to efficiently identify medicinal species. The herbal industry should adopt DNA barcoding to authenticate the raw materials used to manufacture its products.

DNA barcoding can be easily implemented and will play an increasingly important role in medicinal identification because of its ability to rapidly evaluate samples from leaves, seeds, flowers, dry materials, museum specimens, powders or medicinal materials from which DNA can be obtained. DNA barcoding and next-generation sequencing technology are powerful tools for identifying herbal ingredients in patient medicines^43^,^44^. There are limitations to the four common methods of identification, namely, original, microscopy, morphological, and physicochemical identification. The DNA barcoding tool can provide supplementary information to improve classifications and to enable a critical examination of the precision of the four common methods used in medicinal material identification. Descriptions of “medicinal materials” in the pharmacopoeia of China with attached DNA sequences should be actively encouraged. Identification approaches that integrate DNA barcoding, morphological characters and chemical attribute information will achieve maximum efficiency for medicinal material identification. Researchers will have easy access to all the related herbal information in the database. With the development of pyrosequencing, sequencing costs have been dramatically reduced, which opens the way to the high-throughput sequencing of ITS2 sequences, facilitating a wide range of research possibilities using medicinal species. However, for some closely related species, such as the 9 unidentified samples in this study, identification will be very difficult when using universal primers, in which case a better approach would be to use the whole chloroplast genome as a super barcode^45^,^46^.

In conclusion, the current TCM markets are unregulated. The consideration of simple and low-cost measures, such as DNA barcoding, has the potential to make a major contribution to the detection of adulterant products in TCM markets. The present work effectively demonstrates the feasibility of this approach. According to the TCMD, 4.2% of the samples we evaluated were adulterants. The TCMD provides users with easy access for sequence comparisons. The improvement of the TCMD will fulfil its important role in the authentication of medicinal ingredients, which will be beneficial to the entire Chinese herbal industry.

## Materials and Methods

### Plant materials

**Figure 4:**
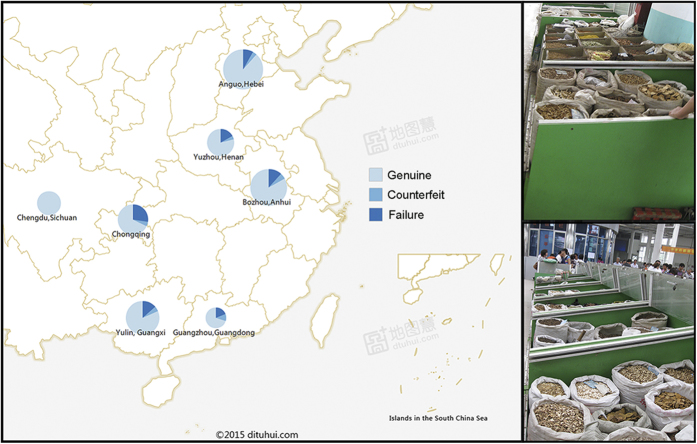
The 7 primary herb markets distributed throughout China. Note: The three colours represent the rate of genuine, adulterant and failed identification for different markets, respectively.

A total of 1436 raw herb samples representing 295 medicinal species were used, including 515 samples of radix et rhizoma, 451 samples of fruit and seeds, 115 samples of herbs, 98 varieties of flos, 82 stem samples, 93 cortex samples, 59 folium samples and 23 fungus samples. The samples were purchased from 7 of the primary herbal markets in China, with 163 samples from Guangxi Yulin (GX), 536 samples from Hebei Anguo (AG), 95 samples from Henan Yuzhou (HN), 402 samples from Anhui Bozhou (BZ), 146 samples from Chongqing Cuqimeng (CQ), 37 samples from Guangdong Qingping (QP) and 57 samples from Sichuan Hehuachi (HUC) (Fig. 4). Of the 295 medicinal species, 294 were listed in the Chinese Pharmacopoeia, and they accounted for approximately 96.4% (133 varieties) of the commonly used varieties in TCM (total of 138 varieties). Thus, the number of samples collected was large enough to be representative. All the specimens were deposited in the herbarium at the Institute of Medicinal Plant Development. The entire list of 1436 samples can be found in Supplementary Table S1 online. The locations of the 7 markets are shown in Fig. 4, which was created using an open source web site (http://www.dituhui.com/) with the latitude and longitude information for the 7 herb markets. The photographs were obtained from AG, which is the largest market in China, and were taken by co-author Baosheng Liao. The map and photographs were combined with Photoshop software.

### DNA extraction and polymerase chain reaction (PCR) amplification

A 75% alcohol solution was used to clean the surfaces of the herbal material prior to DNA extraction to prevent fungal DNA contamination, and then one piece of each sample was ground into powder with a FastPrep bead mill (Retsch MM400, Germany). Total DNA was extracted with a Plant Genome DNA Kit (Tiangen Biotech Co., China), which is based on the CTAB approach. The key procedure was modified as follows. First, the powder was washed with wash buffer three times to remove sticky residues from the precipitant before extraction. Second, after the extraction buffer was added, the samples were incubated at 58 °C for 8–12 hours. Third, an equal amount of ice-cold isopropanol was used to precipitate the DNA at −20 °C in a refrigerator for at least 30 minutes. Other procedures were routinely performed as indicated in the CTAB method. The ITS2 was amplified using universal primers^28^. The PCR reaction mixture consisted of 1 μL (approximately 30 ng) genomic DNA, 1 × PCR buffer without MgCl_2_, 2.0 mM MgCl_2_, 0.2 mM of each dNTP, 0.1 μM of each primer (which were synthesized by Sangon Co., China), and 1.0 U of Taq DNA Polymerase (BiocolorBioScience & Technology Co., China). The PCR conditions were 40 cycles at 94 °C for 30 s, 56 °C for 30 s and 72 °C for 45 s. The entire PCR process was ended by incubating the samples at 72 °C for 10 min with a Peltier Thermal Cycler PTC0200 (Bio-Rad Lab, Inc., USA).

### Sequencing and analysis

The PCR products were purified with a QIAquick PCR purification kit (Tiangen Biotech, Beijing, China) and were directly sequenced on an ABI 3730XL sequencer (Applied Biosystems, USA) by using the original amplification primer as the sequencing primer. The original forward and reverse sequences were assembled with a CodonCode Aligner 3.0. The assembled sequences were annotated and delimited with a hidden Markov model (HMM)-based method^47^, and the complete ITS2 sequences were pasted into the identification module on TCMD (http://www.tcmbarcode.cn/en/). After the query sequence was submitted, a BLASTN algorithm was activated, and its nearest neighbours to all the reference sequences were made available. When a best match to a reference sequence has been found, the identification module can provide a species-level identification and the Latin name of the best-match species will be given^29^.

## Additional Information

**How to cite this article**: Han, J. *et al.* An authenticity survey of herbal medicines from markets in China using DNA barcoding. *Sci. Rep.* **6**, 18723; doi: 10.1038/srep18723 (2016).

## Supplementary Material

Supplementary Table S1

## Figures and Tables

**Table 1:** Identification of adulterant medicinal plant materials.

Sample No.	Latin Name of Medicinal materials	Chinese Name	Latin Name of original species	Medicinal Parts	Markets	Identification Result
FHCQ076	Abri Herba	Jigucao	*Abrus cantoniensis*	Herb	CQ	*Abrus mollis*
S1068	Abri Herba	Jigucao	*Abrus cantoniensis*	Herb	HN	*Abrus mollis*
FHCQ072	Acanthopanacis Cortex	Wujiapi	*Acanthopanax gracilistylus*	Cortex	CQ	*Acanthopanax giraldii*
FHYL237	Acanthopanacis Cortex	Wujiapi	*Acanthopanax gracilistylus*	Cortex	GX	*Periploca sepium*
S0471	Acanthopanacis Cortex	Wujiapi	*Acanthopanax gracilistylus*	Cortex	BZ	*Periploca sepium*
S1188	Acanthopanacis Senticosi Radix et Rhizoma Seu Caulis	Ciwujia	*Acanthopanax senticosus*	Radix et Rhizome	HN	*Alangium chinense*
S0711	Acanthopanacis Senticosi Radix et Rhizoma Seu Caulis	Ciwujia	*Acanthopanax senticosus*	Radix et Rhizome	BZ	*Aralia* sp.
FHCQ065	Acori Tatarinowii Rhizoma	Shichangpu	*Acorus tatarinowii*	Radix et Rhizome	CQ	*Acorus* sp.
S0206	Acori Tatarinowii Rhizoma	Shichangpu	*Acorus tatarinowii*	Radix et Rhizome	BZ	*Acorus* sp.
FHAG014	Acori Tatarinowii Rhizoma	Shichangpu	*Acorus tatarinowii*	Radix et Rhizome	AG	*Acorus calamus*
FHAG234	Acori Tatarinowii Rhizoma	Shichangpu	*Acorus tatarinowii*	Radix et Rhizome	AG	*Acorus calamus*
FHAG420	Albiziae Flos	Hehuanhua	*Albizia julibrissin*	Flowers	AG	*Celastrus orbiculatus*
FHYL134	Albiziae Cortex	Hehuanpi	*Albizia julibrissin*	Cortex	GX	*Albizia kalkora*
FHYL440	Albiziae Cortex	Hehuanpi	*Albizia julibrissin*	Cortex	GX	*Albizia kalkora*
FHCQ187	Albiziae Cortex	Hehuanpi	*Albizia julibrissin*	Cortex	CQ	*Albizia kalkora*
AG015	Albiziae Cortex	Hehuanpi	*Albizia julibrissin*	Cortex	AG	*Albizia kalkora*
S0636	Albiziae Cortex	Hehuanpi	*Albizia julibrissin*	Cortex	BZ	*Albizia kalkora*
FHAG163	Alpiniae Oxyphyllae Fructus	Yizhi	*Alpinia oxyphylla*	Fruits & Seeds	AG	*Foeniculum vulgare*
S0243	Angelicae Dahuricae Radix	Baizhi	*Angelica dahurica var.formosana*	Radix et Rhizome	BZ	*Abrus pulchellus*
S0365	Angelicae Pubescentis Radix	Duhuo	*Angelica pubescens* f. *biserratn*	Radix et Rhizome	BZ	*Angelica amurensis*
S0485	Angelicae Pubescentis Radix	Duhuo	*Angelica pubescens* f. *biserratn*	Radix et Rhizome	BZ	*Levisticum officinale*
AG165	Arnebiae Radix	Zicao	*Arnebia euchroma*	Radix et Rhizome	AG	*Lithospermum erythrorhizon*
FHYL175	Bupleuri Radix	Chaihu	*Bupleurum chinense*	Radix et Rhizome	GX	*Bupleurum* sp.
S0552	Bupleuri Radix	Chaihu	*Bupleurum chinense*	Radix et Rhizome	BZ	*Bupleurum* sp.
FHYL394	Bupleuri Radix	Chaihu	*Bupleurum chinense*	Radix et Rhizome	GX	*Bupleurum* sp.
S0511	Bupleuri Radix	Chaihu	*Bupleurum chinense*	Radix et Rhizome	BZ	*Bupleurum* sp.
FHCQ015	Dalbergiae Odoriferae Lignum	Jiangxiang	*Dalbergia odorifera*	Stems	CQ	*Caesalpinia sappan*
AG008	Dalbergiae Odoriferae Lignum	Jiangxiang	*Dalbergia odorifera*	Stems	AG	*Caesalpinia sappan*
S0219	Dalbergiae Odoriferae Lignum	Jiangxiang	*Dalbergia odorifera*	Stems	BZ	*Caesalpinia sappan*
S0410	Dalbergiae Odoriferae Lignum	Jiangxiang	*Dalbergia odorifera*	Stems	BZ	*Caesalpinia sappan*
S1241	Dalbergiae Odoriferae Lignum	Jiangxiang	*Dalbergia odorifera*	Stems	QP	*Caesalpinia sappan*
S1245	Dalbergiae Odoriferae Lignum	Jiangxiang	*Dalbergia odorifera*	Stems	QP	*Caesalpinia sappan*
FHYL083	Erycibes Caulis	Dinggongteng	*Erycibe schmidtii*	Stems	GX	Covolvulaceae
AG230	Euphorbia Ebracteolatae Radix	Langdu	*Euphorbia fischeriana*	Radix et Rhizome	AG	*Stellera chamaejasme*
S0082	Sojae Semen Praeparatum	Dandouchi	*Glycine max*	Fruits & Seeds	BZ	*Phaseolus vulgaris*
FHYL133	Inulae Flos	Xuanfuhua	*Inula japonica*	Flowers	GX	*Inula lineariifolia*
S1135	Inulae Flos	Xuanfuhua	*Inula japonica*	Flowers	HN	*Inula lineariifolia*
S0612	Inulae Flos	Xuanfuhua	*Inula japonica*	Flowers	BZ	*Inula lineariifolia*
S0757	Inulae Flos	Xuanfuhua	*Inula japonica*	Flowers	BZ	*Inula lineariifolia*
FHYL059	Lonicerae Japonicae Flos	Jinyinhua	*Lonicera japonica*	Flowers	GX	*Lonicera macranthoides*
AG069	Lonicerae Japonicae Flos	Jinyinhua	*Lonicera japonica*	Flowers	AG	*Lonicera macranthoides*
FHCQ151	Moslae Herba	Xiangru	*Mosla chinensis*	Herb	CQ	*Elsholtzia ciliata*
MM022	Ginseng Radix et Rhizoma	Renshen	*Panax ginseng*	Radix et Rhizome	AG	*Panax quinquefolium*
MM022-1	Ginseng Radix et Rhizoma	Renshen	*Panax ginseng*	Radix et Rhizome	AG	*Panax quinquefolium*
SQAG02	Ginseng Radix et Rhizoma	Renshen	*Panax ginseng*	Radix et Rhizome	AG	*Panax quinquefolium*
AG264	Panacis Japonici Rhizoma	Zhujieshen	*Panax japonicus*	Radix et Rhizome	AG	*Panax* sp.
S0593	Pseudolaricis Cortex	Tujingpi	*Pseudolarix amabilis*	Cortex	BZ	*Celastrus angulatus*
AG104	Pseudolaricis Cortex	Tujingpi	*Pseudolarix amabilis*	Cortex	AG	*Celastrus angulatus*
FHCQ123	Puerariae Thomsonii Radix	Fenge	*Pueraria thomsonii*	Radix et Rhizome	CQ	*Pueraria lobata*
S0087	Pulsatillae Radix	Baitouweng	*Pulsatilla chinensis*	Radix et Rhizome	BZ	*Platycodon grandiflorus*
S0669	Rhapontici Radix	Loulu	*Rhaponticum uniflorum*	Radix et Rhizome	BZ	*Echinops latifolius*
B1	Rubi Rhizoma	Maomeigen	*Rubus parvifolius*	Radix et Rhizome	BZ	*Cirsium japonicum*
B2	Rubi Rhizoma	Maomeigen	*Rubus parvifolius*	Radix et Rhizome	BZ	*Cirsium japonicum*
B3	Rubi Rhizoma	Maomeigen	*Rubus parvifolius*	Radix et Rhizome	BZ	*Cirsium japonicum*
B4	Rubi Rhizoma	Maomeigen	*Rubus parvifolius*	Radix et Rhizome	BZ	*Cirsium japonicum*
CZG01	Rubi Rhizoma	Maomeigen	*Rubus parvifolius*	Radix et Rhizome	AG	*Rubus alceaefolius*
CZG02	Rubi Rhizoma	Maomeigen	*Rubus parvifolius*	Radix et Rhizome	AG	*Rubus alceaefolius*
AG1	Rubi Rhizoma	Maomeigen	*Rubus parvifolius*	Radix et Rhizome	AG	*Rosa chinensis*
AG3	Rubi Rhizoma	Maomeigen	*Rubus parvifolius*	Radix et Rhizome	AG	*Rosa chinensis*
AG4	Rubi Rhizoma	Maomeigen	*Rubus parvifolius*	Radix et Rhizome	AG	*Rosa chinensis*
AG2	Rubi Rhizoma	Maomeigen	*Rubus parvifolius*	Radix et Rhizome	AG	*Rosa chinensis*
S1297	Spirodelae Herba	Fuping	*Spirodela polyrrhiza*	Herb	HN	*Wolffia globosa*

Note: BZ represent Anhui Bozhou Herb Market; AG represent Hebei Anguo Herb Market; CQ represent Chongqing Chuqimen Herb Market; GX represent Guangxi Yulin Herb Market; HN represent Henan Yuzhou Herb Market; QP represent Guangdong Qingping Herb Market; HUC represent Sichuan Hehuachi Herb Market.

## References

[b1] Chan T. Y. Incidence of Herb-Induced Aconitine Poisoning in Hong Kong. Drug Saf. 25, 823–828 (2002). 12222992 10.2165/00002018-200225110-00006

[b2] Lowe L., Matteucci M. J. & Schneir A. B. Herbal aconite tea and refractory ventricular tachycardia. N. Engl. J. Med. 353, 1532–1532 (2005). 16207864 10.1056/NEJMc051568

[b3] Poon W. T. *et al.* Aconite poisoning in camouflage. Hong Kong Med. J. 12, 456–459 (2006). 17148799

[b4] Fotiou F., Aravind S., Wang P. & Nerapusee O. Impact of illegal trade on the quality of epoetin alfa in Thailand. Clin. Ther. 31, 336–346 (2009). 19302906 10.1016/j.clinthera.2009.02.014

[b5] Gautam C., Utreja A. & Singal G. Spurious and counterfeit drugs: a growing industry in the developing world. Postgrad. Med. J. 85, 251–256 (2009). 19520877 10.1136/pgmj.2008.073213

[b6] Organization, W. H. *Medicines: spurious/falsely-labelled/falsified/counterfeit (SFFC) medicines.* (2012), Available at: http://www.who.int/mediacentre/factsheets/fs275/en/. (Date of access: 02/01/2015).

[b7] Garuba H. A., Kohler J. C. & Huisman A. M. Transparency in Nigeria’s public pharmaceutical sector: perceptions from policy makers. Global Health 5, 14 (2009). 19874613 10.1186/1744-8603-5-14 PMC2775729

[b8] Dégardin K., Roggo Y. & Margot P. Understanding and fighting the medicine counterfeit market. J. Pharm. Biomed. Anal. 87, 167–175 (2014). 23384475 10.1016/j.jpba.2013.01.009

[b9] Marini R. *et al.* Reliable low-cost capillary electrophoresis device for drug quality control and counterfeit medicines. J. Pharm. Biomed. Anal. 53, 1278–1287 (2010). 20719445 10.1016/j.jpba.2010.07.026

[b10] Busse W. The significance of quality for efficacy and safety of herbal medicinal products. Drug Inf. J. 34, 15–23 (2000).

[b11] De Smet P. A. Health risks of herbal remedies: an update. Clin. Pharmacol. Ther. 76, 1–17 (2004). 15229459 10.1016/j.clpt.2004.03.005

[b12] Sperl W. *et al.* Reversible hepatic veno-occlusive disease in an infant after consumption of pyrrolizidine-containing herbal tea. Eur. J. Pediatr. 154, 112–116 (1995). 7720737 10.1007/BF01991912

[b13] Johanns E. S. *et al.* [An epidemic of epileptic seizures after consumption of herbal tea]. Ned. Tijdschr. Geneeskd. 146, 813–816 (2002). 12014242

[b14] Chen S. P. L. *et al.* Aconite Poisoning over 5 Years. Drug Saf. 35, 575–587 (2012). 22631223 10.2165/11597470-000000000-00000

[b15] Phua D., Cham G. & Seow E. Two instances of Chinese herbal medicine poisoning in Singapore. Singapore Med. J. 49, e131–133 (2008). 18465037

[b16] Dondorp A. *et al.* Fake antimalarials in Southeast Asia are a major impediment to malaria control: multinational cross-sectional survey on the prevalence of fake antimalarials. Trop. Med. Int. Health 9, 1241–1246 (2004). 15598255 10.1111/j.1365-3156.2004.01342.x

[b17] Lee S. *et al.* Fanconi’s syndrome and subsequent progressive renal failure caused by a Chinese herb containing aristolochic acid. Nephrology 9, 126–129 (2004). 15189173 10.1111/j.1440-1797.2003.00232.x

[b18] Lo S. H. K. *et al.* Aristolochic acid nephropathy complicating a patient with focal segmental glomerulosclerosis. Nephrol. Dial. Transplant. 19, 1913–1915 (2004). 15199198 10.1093/ndt/gfh159

[b19] Vanherweghem J. L. *et al.* Rapidly progressive interstitial renal fibrosis in young women: association with slimming regimen including Chinese herbs. Lancet 341, 387–391 (1993). 8094166 10.1016/0140-6736(93)92984-2

[b20] Xin T. *et al.* Survey of commercial Rhodiola products revealed species diversity and potential safety issues. Sci. Rep. 5, 8337, doi: 10.1038/srep08337 (2015). 25661009 PMC4321177

[b21] Panusa A., Multari G., Incarnato G. & Gagliardi L. High-performance liquid chromatography analysis of anti-inflammatory pharmaceuticals with ultraviolet and electrospray-mass spectrometry detection in suspected counterfeit homeopathic medicinal products. J. Pharm. Biomed. Anal. 43, 1221–1227 (2007). 17127029 10.1016/j.jpba.2006.10.012

[b22] Puchert T., Lochmann D., Menezes J. & Reich G. Near-infrared chemical imaging (NIR-CI) for counterfeit drug identification—a four-stage concept with a novel approach of data processing (Linear Image Signature). J. Pharm. Biomed. Anal. 51, 138–145 (2010). 19766424 10.1016/j.jpba.2009.08.022

[b23] Lindegårdh N., Dondorp A., Singhasivanon P., White N. & Day N. Validation and application of a liquid chromatographic-mass spectrometric method for determination of artesunate in pharmaceutical samples. J. Pharm. Biomed. Anal. 45, 149–153 (2007). 17553648 10.1016/j.jpba.2007.04.030

[b24] Hebert P. D. N., Cywinska A., Ball S. L. & deWaard J. R. Biological identifications through DNA barcodes. Proc. R. Soc. London, Ser. B 270, 313–321 (2003). 10.1098/rspb.2002.2218 PMC1691236 12614582

[b25] Techen N., Parveen I., Pan Z. & Khan I. A. DNA barcoding of medicinal plant material for identification. Curr. Opin. Biotechnol. 25, 103–110 (2014). 24484887 10.1016/j.copbio.2013.09.010

[b26] Hebert P. D. & Gregory T. R. The promise of DNA barcoding for taxonomy. Syst. Biol. 54, 852–859 (2005). 16243770 10.1080/10635150500354886

[b27] Miller S. E. DNA barcoding and the renaissance of taxonomy. Proc. Natl. Acad. Sci. USA 104, 4775–4776 (2007). 17363473 10.1073/pnas.0700466104 PMC1829212

[b28] Chen S. *et al.* Validation of the ITS2 region as a novel DNA barcode for identifying medicinal plant species. PLoS One 5, e8613 (2010). 20062805 10.1371/journal.pone.0008613 PMC2799520

[b29] Chen S. *et al.* A renaissance in herbal medicine identification: from morphology to DNA. Biotechnol. Adv. 32, 1237–1244 (2014). 25087935 10.1016/j.biotechadv.2014.07.004

[b30] Administration T. K. F. a. D. The Korean Pharmacopoeia. 10th ed., (KFDA press, 2012).

[b31] Commission, E. P. European Pharmacopoeia. 8th ed., (Directorate for the Quality of Medicines & HealthCare of the Council of Europe (EDQM), 2013).

[b32] Committee, J. P. E. Japanese Pharmacopoeia. 16th ed., (The Ministry of Health, Labour and Welfare, 2011).

[b33] Committee, N. P. Chinese Pharmacopoeia. 2010th ed., (China Medical Science Press, 2010).

[b34] Convention, U. S. P. U.S. Pharmacopeia 36/National Formulary 31. (U.S. Pharmacopeial Convention Incorporation, 2013).

[b35] IP, C. Indian Pharmacopoeia. 2010th ed., (The Indian Pharmacopoeia Commission, 2010).

[b36] Han J. *et al.* The short ITS2 sequence serves as an efficient taxonomic sequence tag in comparison with the full-length ITS. BioMed Res. Int. 2013 (2013). 10.1155/2013/741476 PMC3581084 23484151

[b37] Kool A. *et al.* Molecular identification of commercialized medicinal plants in Southern Morocco. PloS one 7, e39459 (2012). 22761800 10.1371/journal.pone.0039459 PMC3384669

[b38] Ross H. A., Murugan S. & Li W. L. S. Testing the reliability of genetic methods of species identification via simulation. Systematic biology 57, 216–230 (2008). 18398767 10.1080/10635150802032990

[b39] Han J. *et al.* Relationship between DNA Barcoding and Chemical Classification of Salvia Medicinal Herbs. Chin. Herb. Med. 2, 16–29 (2010).

[b40] Espinoza E. O., Mann M. J. & Bleasdell B. Arsenic and mercury in traditional Chinese herbal balls. N. Engl. J. Med. 333, 803–804 (1995). 7643901 10.1056/NEJM199509213331217

[b41] Natori S. Application of herbal drugs to health care in Japan. J. Ethnopharmacol. 2, 65–70 (1980). 7464186 10.1016/0378-8741(80)90032-x

[b42] Liu C. *et al.* DNA barcode goes two-dimensions: DNA QR code web server. PloS one 7, e35146 (2012). 22574113 10.1371/journal.pone.0035146 PMC3344831

[b43] Cheng X. *et al.* Biological ingredient analysis of traditional Chinese medicine preparation based on high-throughput sequencing: the story for Liuwei Dihuang Wan. Sci. Rep. 4, 5147 (2014). 24888649 10.1038/srep05147 PMC4042125

[b44] Coghlan M. L. *et al.* Deep sequencing of plant and animal DNA contained within traditional Chinese medicines reveals legality issues and health safety concerns. PLoS Genet. 8, e1002657 (2012). 22511890 10.1371/journal.pgen.1002657 PMC3325194

[b45] Chen X., Li Q., Li Y., Qian J. & Han J. Chloroplast genome of Aconitum barbatum var. puberulum (Ranunculaceae) derived from CCS reads using the PacBio RS platform. Front. Plant Sci. 6, 42 (2015). 25705213 10.3389/fpls.2015.00042 PMC4319492

[b46] Li Q. *et al.* High–accuracy *de novo* assembly and SNP detection of chloroplast genomes using a SMRT circular consensus sequencing strategy. New Phytol. 204, 1041–1049 (2014). 25103547 10.1111/nph.12966

[b47] Keller A. *et al.* 5.8 S-28S rRNA interaction and HMM-based ITS2 annotation. Gene 430, 50–57 (2009). 19026726 10.1016/j.gene.2008.10.012

